# A key for the determination of European species of *Eosentomon* Berlese, 1909 (Protura, Eosentomata, Eosentomidae)

**DOI:** 10.3897/zookeys.742.22664

**Published:** 2018-03-12

**Authors:** Julia Shrubovych, Ernest C. Bernard

**Affiliations:** 1 Institute of Soil Biology, Biology Centre Czech Academy of Sciences, Na Sádkách 7, 370 05, České Budějovice, Czech Republic; 2 Institute of Systematics and Evolution of Animals, Polish Academy of Sciences, ul. Sławkowska 17, 31-016 Krakow, Poland; 3 State Museum of Natural History, Ukrainian Academy of Sciences, Teatral’na St. 18, UA 79008 L’viv, Ukraine; 4 University of Tennessee, Entomology and Plant Pathology, 2505 E.J. Chapman Drive, 370 Plant Biotechnology, Knoxville, TN 37996, USA

**Keywords:** Europe, *Eosentomon*, Protura, taxonomic key

## Abstract

European species of *Eosentomon* are examined. A taxonomic key to identification of 61 *Eosentomon* species is provided based on body chaetotaxy, shape, and position of sensilla on the foretarsus and shape of sensilla on the maxillary palps. Biogeographically, 13 of the known European *Eosentomon* species are known only from their type localities.

## Introduction

The proturan genus *Eosentomon* Berlese, 1909 has a worldwide distribution and contains approximately 310 described species (Szeptycki 2007, Bu and Yin 2007, Shrubovych and Szeptycki 2008, Nakamura and Likhitrakarn 2009, Nakamura 2010), of which 61 have been described from Europe (Szeptycki 2007, Shrubovych and Szeptycki 2008). A key for the identification of European *Eosentomon* species was created by Nosek (1973) for 14 species, in which the author differentiated species into four groups according to the shape of female squama genitalis. This approach followed Tuxen (1960), who divided worldwide *Eosentomon* into 11 groups based on the squama genitalis. Szeptycki (1984, 1985a, 1985b, 1986) wrote a series of papers with keys to identification of four groups of Polish *Eosentomon* species, in which cephalic chaetotaxy was used for group separation (Szeptycki, 1986). Both Nosek and Szeptycki frequently used in their keys the shape of the female squama genitalis, which made identification of males and young stages impossible except by association. The present paper contains an identification key to European *Eosentomon* species based primarily on chaetotaxy, shape, and position of sensilla on the foretarsus and the shape of sensilla on the maxillary palpi.

## Materials and methods

Type materials were examined of 31 *Eosentomon* species deposited in the collection of Prof. Szeptycki in the Institute of Systematics and Evolution of Animals PAS, eight *Eosentomon* species in the collection of J. Rusek in the Institute of Soil Biology BCCAS and one species deposited in the collection the State Museum of Natural History NASU. Information about the taxonomy of other *Eosentomon* species was taken from original descriptions or redescriptions of type materials in Tuxen (1964), Nosek (1973) and various other papers. Head chaetotaxy is labelled as in Szeptycki (1984), and body chaetotaxy is labeled according to Bernard (1990).

The geographical distribution of these species is given according to recent published data (Szeptycki 2007, Shrubovych 2010, Christian 2011, Galli et al. 2011, Blesić and Mitrovski-Bogdanović 2012, Shrubovych and Sterzyńska 2015, Shrubovych et al. 2015, Shrubovych and Fiera 2016). Each species was assigned to a major biogeographic region (Alpine, Boreal, Continental, Pannonian, Mediterranean, Macaronesian) according to the European Environment Commission (2017) Natura 2000 terminology of European biogeographic regions (see map in: https://www.eea.europa.eu/data-and-maps/figures/biogeographical-regions-in-europe-2).

## Results and discussion

Taxonomic characters used in the key are present in juvenile stages as well as the adults. The shapes of the parts of the adult female squama genitalis may have great phylogenetic value and can serve as additional characters for identification of species. The characters used in the key, such as shape of maxillary palpi, chaetotaxy of the head, shape and position of sensilla on the foretarsus and position of seta *P1a* on tergite VII are stable from the second juvenile stage (larva I) (Nosek 1973, Szeptycki 1965b). All setae on the notal tergites and on the abdominal segments are present from the maturus junior stage (Imadaté 1965).

Analysis of the geographical distribution of European *Eosentomon* shows that nearly all species have been collected only in Europe, except for two (*E. delicatum* and *E. mixtum*) that have also been recorded from northern Africa. The majority of the species have been recorded only from Central Europe, probably due to many years of active work in this region by Josef Nosek, Josef Rusek and Andrzej Szeptycki. Sixteen species are known from Western Europe and only two species have been reported from Eastern Europe (Table 1). Nineteen species occur in Southern Europe and only two species have been noted from Northern Europe. If intensity of collection and study is correlated with number of recognized species then there are more species yet to be discovered in Europe. Thirteen of the 61 species are known only from their type localities: therefore, it is difficult to assert endemicity within such a poorly studied group of microarthropods. Nevertheless, two species could be endemics of the East Carpathians. The occurrence of *E. carpaticum* has been confirmed in the Carpathian Mountains of Ukraine, Romania, Hungary (Shrubovych and Sterzyńska 2015, Shrubovych et al. 2015) and in the Slovakian Carpathians (unpublished data). Therefore, it can be considered an Eastern-Carpathian endemic (Table 1). The collection of *E. carpaticum* in the lowlands of the Transcarpathian region is consistent with an earlier report that this species has a wide ecological plasticity and environmental distribution pattern, and can predominate in Protura communities outside of mountain habitats in azonal habitats, such as floodplain forests (Sterzyńska et al. 2012). A similar situation exists with *E. enigmaticum*, which was collected in the Ukrainian and Slovakian Carpathians (Shrubovych and Sterzynska 2015, unpublished data) and has been considered an Eastern-Carpathian endemic (Shrubovych and Sterzynska 2017); however, in Poland this species was found outside of mountainous habitats.

**Table 1.** Distribution pattern of *Eosentomon* species in European biogeographical regions.

Species	Countries	Biogeographical regions
*E. armatum* Stach, 1927	Nearly all of Europe	Continental-Pannonian-Mediterranean
*E. bloszyki* Szeptycki, 1985	Poland, Czech Republic, Luxembourg, Germany, Austria, Ukraine	Continental
*E. boedvarssoni* Nosek, 1973	Sweden	Boreal
*E. bohemicum* Rusek, 1966	Czech Republic, Poland	Continental
*E. briophillum* Szeptycki, 1986	Poland, type locality only	Continental
*E. canarinum* Szeptycki, 2004	Canary Islands	Macaronesian
*E. carolae* Condé, 1947	France, Spain	Mediterranean
*E. carpaticum* Szeptycki, 1985	Poland, Ukraine, Romania, Hungary, Slovakia	Alpine-Continental
*E. cetium* Szeptycki & Christian, 2000	Austria, type locality only	Continental
*E. coiffaiti* Condé, 1961	Minorca, Serbia	Macaronesian-Continental
*E. condei* da Cunha, 1950	Portugal, Spain	Mediterranean
*E. delicatum* Gisin, 1945	All Europe, North Africa	Alpine-Boreal-Continental-Pannonian-Mediterranean
*E. denisi* Condé, 1947	France, Spain	Mediterranean
*E. enigmaticum* Szeptycki, 1986	Poland, Ukraine, Romania, Slovakia	Alpine-Continental
*E. fichteliense* Rusek, 1988	Germany, type locality only	Continental
*E. foliaceus* Rusek, 1988	Germany, Poland	Continental
*E. foroiuliense* Torti & Nosek, 1984	Italy, type locality only	Continental
*E. funkei* Rusek, 1988	Germany, Luxembourg	Continental
*E. gamae* Aldaba, 1986	Portugal, type locality only	Mediterranean
*E. germanicum* Prell, 1912	Nearly all of Europe, Morocco	Alpine-Boreal-Continental-Mediterranean
*E. gisini* Nosek, 1967	Austria, Slovakia	Alpine
*E. gramineum* Szeptycki, 1986	Poland, Ukraine	Continental
*E. kamenickiense* Rusek, 1974	Czech Republic, type locality only	Continental
*E. longisquamum* Szeptycki, 1986	Poland, Austria	Continental
*E. lusitanicum* Aldaba, 1986	Portugal, type locality only	Mediterranean
*E. luxembourgense* Szeptycki, 2001	Luxembourg, Austria	Continental
*E. mariae* Szeptycki, 1986	Poland, Austria, Germany, Luxembourg, Ukraine	Continental
*E. mirabile* Szeptycki, 1984	Poland, Germany, Austria, Canary Islands, France, Ukraine	Continental-Macaronesian
*E. mixtum* Condé, 1945	France, Austria, Czech Republic, Germany, Slovakia, Madeira	Alpine-Continental-Macaronesian
*E. noseki* Tuxen, 1982	Macaronesia, Spain, Italy	Macaronesian-Mediterranean
*E. occidentale* Szeptycki, 1985	Poland	Continental
*E. palustre* Szeptycki & Sławska, 2000	Poland, type locality only	Continental
*E. parvum* Szeptycki, 1986	Austria, Poland	Continental
*E. pastorale* Szeptycki, 2001	Austria, Luxembourg	Continental
*E. paucrum* Szeptycki, 2001	Luxembourg	Continental
*E. pinetorum* Szeptycki, 1984	Austria, Czech Republic, Poland, Ukraine, Romania	Alpine-Continental
*E. pinkyae* Arbea-Polite, 1990	Spain, type locality only	Mediterranean
*E. polonicum* Szeptycki, 1985	Poland	Continental
*E. posnaniense* Szeptycki, 1986	Poland, Austria	Continental
*E. pratense* Rusek, 1973	Czech Republic, Poland, former Yugoslavia, Slovakia, Germany, Austria, Ukraine	Continental
*E. rafalskii* Szeptycki, 1985	Poland, Czech Republic, Germany	Continental
*E. romanum* Nosek, 1969	Italy	Mediterranean-Continental
*E. rusekianum* Stumpp & Szeptycki, 1989	Germany, Austria, Poland	Continental
*E. scytha* Shrubovych & Szeptycki, 2008	Ukraine, type locality only	Continental
*E. semiarmatum* Szeptycki, 1986	Balearic Islands, France, Germany, Poland, Ukraine, Romania	Mediterranean-Continental
*E. sexsetosum* Szeptycki, 1985	Luxembourg, Poland	Continental
*E. silesiacum* Szeptycki, 1985	Germany, Czech Republic, Poland, Luxembourg, Sweden	Boreal-Continental
*E. silvaticum* Szeptycki, 1986	Poland, Luxembourg, Romania	Alpine-Continental
*E. solarzi* Szeptycki, 1993	European part of Russia, type locality only	Continental
*E. stachi* Rusek, 1966	Austria, Luxembourg, Poland, Slovakia, Ukraine, Romania	Alpine - Continental
*E. stompi* Szeptycki & Weiner, 1993	Germany, Luxembourg	Continental
*E. stumppi* Rusek, 1988	Germany, Austria	Continental
*E. sudeticum* Szeptycki, 1985	Poland, Czech Republic	Continental
*E. transitorium* Berlese, 1909	all Europe	Alpine-Boreal-Continental-Pannonian-Mediterranean
*E. ulinense* Szeptycki, 1999	Poland	Continental
*E. umbrosum* Szeptycki, 2001	Luxembourg	Continental
*E. vindobonense* Szeptycki & Christian, 2000	Austria, type locality only	Continental
*E. vulgare* Szeptycki, 1984	Poland, Czech Republic, Germany, Austria, Luxembourg, Ukraine	Continental
*E. wanda* Szeptycki, 1985	Poland, type locality only	Continental
*E. weinerae* Szeptycki, 2001	Austria, Luxembourg	Continental
*E. zodion* Szeptycki, 1985	Poland, Ukraine	Continental

The existence of at least 61 European *Eosentomon* species, with only two of its species outside the continent (Mediterranean Africa), strongly suggests that the remaining continents must have many more species than are currently known from them. All of the world’s *Eosentomon* species have ranges restricted to a single continent, and most are apparently specialized to a particular biome or specialized habitat. Much more collecting needs to be done, even in Europe, for us to understand the diversity of these enigmatic hexapods.

### Key to the European *Eosentomon* species

**Table:** 

1	Tergite VII with 2 *A*-setae (seta *A4*)	**2**
–	Tergite VII with greater number of *A*-setae	**3**
2	Tergite IV with 8 *A*-setae (*A5* absent), foretarsal sensillum *a* clearly shorter than *c* (see Rusek 1974: figs 1, 2)	***E. kamenickiense***
–	Tergite IV with 10 *A*-setae (*A5* present), foretarsal sensilla *a* and *c* equal in length (see Rusek 1973: figs 1, 2, 10)	***E. pratense***
3	Tergite VII with 4 *A*-setae (setae *A4*, *A5*)	**4**
–	Tergite VII with other number of *A*-setae	**28**
4	Tergite V with 8 *A*-setae (*A3* absent)	5*
–	Tergite V with 10 *A*-setae (*A3* present)	**16**
5	Head with *aa* and *pa* setal pairs	**6**
–	Head with one pair or without additional setae	**11**
Two species, *E. denisi* and *E. condei*, will key to couplet 5 but their cephalic chaetotaxy is unknown. *Eosentomon denisi* possesses eight *A*-setae on tergites V–VII, the female squama genitalis is of the “*wheeleri*” type (see Nosek 1973: 92–94, fig. 26 I), the foretarsus is 110 μm long. The other species, *E. condei*, has eight *A*-setae on tergites V–VI and six *A*-setae on tergite VII, and the foretarsus length is 80 μm (see Nosek 1973: 107–108).		
6	Seta *P1a* at level of *P2* on tergite VII (see Szeptycki 1985a: fig. 25; Szeptycki and Sławska 2000: fig. 19)	**7**
–	Seta *P1a* posterior to level of *P2*, extending past hind margin of tergite VII	**10**
7	Foretarsal sensillum *d* long, reaching base of *t3* (Szeptycki 1985a: fig. 28; Szeptycki 2001: fig. 61)	**8**
–	Foretarsal sensillum *d* short, reaching base of *α5* (see Szeptycki 2001: fig. 81; Szeptycki and Sławska 2000: fig. 10)	**9**
8	Foretarsal sensillum *c*’ long, base proximal to line *α6 – δ5* (Szeptycki 1985a: fig. 27), length of foretarsus 100–105 μm	***E. bloszyki***
–	Foretarsal sensillum *c*’ short, base distal to line *α6 – δ5* (Szeptycki 2001: fig. 60), length of foretarsus 80–85 μm	***E. paucrum***
9	Foretarsal sensillum *t1* nearer to *α3*’ than to *α3*, rostral and subrostral setae equal in length (see Szeptycki 2001: figs 72, 85), length of foretarsus 105–115 μm	***E. pastorale***
–	Sensillum *t1* midway between *α3* and *α3*’, rostral setae shorter than subrostral setae (see Szeptycki and Sławska 2000: figs 2, 12), foretarsus 70–80 μm	***E. palustre***
10	Foretarsal sensillum *c*’ thick, proximal to line *α6 – δ5* (see Nosek 1973: fig. 36B; Szeptycki 1985a: fig. 48), length of foretarsus 80–95 μm	***E. stachi***
–	Foretarsal sensillum *c*’ slender, on line *α6 – δ5* (see Szeptycki 1985: fig. 85), length of foretarsus 95–100 μm	***E. carpaticum***
11	Head with *pa* setae only, foretarsal sensillum *b’2* very long, foliaceous (see Rusek 1988: figs 1D, 2B), length of foretarsus 95 μm	***E. foliaceus***
–	Head without additional setae, foretarsal sensillum *b’2* shorter, sensilliform	**12**
12	Notal seta *P2a* clearly longer than one-third the length of *P3a*; setae on tergite XI very short, one-sixth the length of those on tergite X (see Szeptycki 1985b: figs 48, 52, 56, 70)	**13**
–	Notal setae *P2a* one-third the length of *P3a*; setae on tergite XI half as long as setae on tergite X (see Szeptycki 1985b: figs 13, 17, 22, 34)	**15**
13	Tergite VI with 8 *A*-setae (*A3* absent) (see Szeptycki 1985b: fig. 39; Szeptycki and Weiner 1993: fig. 12)	**14**
–	Tergite VI with 6 *A*-setae (*A1, A3* absent) (see Szeptycki 1985b: fig. 57)	***E. sexsetosum***
14	Dorsal sensillum on maxillary palpus longer than lateral sensillum, rostral and subrostral setae subequal (see Szeptycki 1985b: fig. 46), length of foretarsus 100–110 μm ***E. occidentale***	***E. occidentale***
–	Sensilla on maxillary palpus equal in length, rostral and subrostral setae subequal (see Szeptycki and Weiner 1993: fig. 5), length of foretarsus 85–100 μm	***E. stompi***
15	Tergite IV with 8 *A*-setae (*A3* absent), dorsal sensillum on maxillary palpus longer than lateral sensillum (see Rusek 1966: fig. 7; Szeptycki 1985b: figs 4, 6)	***E. bohemicum***
–	Tergite IV with 10 *A*-setae, sensilla on maxillary palpus equal in length (see Szeptycki 1985: figs 23, 30)	***E. polonicum***
16	Tergite VI with 10 *A*-setae	**17**
–	Tergite VI with 8 *A*-setae	**18**
17	Foretarsal sensilla *a* and *c* short, sensillum *f2* very short, one-fifth length of *f1* (see Torti and Nosek 1984: fig. 1B), length of foretarsus 112 μm	***E. foroiuliense***
–	Foretarsal sensillum *a* longer than *c*, sensilla *f1* and *f2* nearly equal in length, female squama genitalis with small beak-like terminus (see Arbea-Polite 1990: figs 2a, 11), length of foretarsus 75–85 μm	***E. pinkyae***
18	Seta *P1a* at level of *P2* on tergite VII (see Szeptycki 2001: figs 19, 20)	**19**
–	Seta *P1a* posterior to level of *P2* on tergite VII	**21**
19	Foretarsal sensilla *a* and *c* equal in length, female squama genitalis of “*romanum*” type (see Nosek 1973: figs 38A, J, J’)	***E. romanum***
–	Foretarsal sensillum *a* clearly shorter than *c*, female squama genitalis of “*transitorium*” type (see Szeptycki 2001: fig. 17; Nosek 1973: fig. 33 I)	**20**
20	Foretarsal sensillum *c*’ proximal to base of *α6* or to line *α6 – δ5*, broad (see Szeptycki 2001: figs 4, 12, 14), length of foretarsus 65–75 μm	***E. luxembourgense***
–	Foretarsal sensillum *c*’ to line *α6 – δ5*, slender (see Nosek 1973: fig. 33 A), length of foretarsus 90–100 μm	***E. delicatum***
21	Foretarsal sensillum *c*’ on line *α6 – δ5*	**22**
–	Foretarsal sensillum *c*’ proximal to line *α6 – δ5*	**24**
22	Foretarsal sensillum *t1* nearer to *α3*’ than to *α3*, sensilla on maxillary palpus nearly equal in length (see Szeptycki 1985a: figs 102, 109)	***E. wanda***
–	Foretarsal sensillum *t1* midway between *α3* and *α3*’ or nearer to *α3*, dorsal sensillum on maxillary palpus clearly longer than lateral (see Szeptycki 1985a: figs 122, 125, Shrubovych and Szeptycki 2008: figs 5, 20)	**23**
23	Sensillum *t1* midway between *α3* and *α3*’, rostral and subrostral setae equal in length (Shrubovych and Szeptycki 2008: figs 4, 9)	***E. scytha***
–	Sensillum *t1* much closer to *α3* than to *α3*’, rostral setae slightly shorter than subrostral setae (see Szeptycki 1985a: figs 120, 125)	***E. zodion***
24	Head with *aa* and *pa* setae	**25**
–	Head with *pa* setae only (see Szeptycki 2001: fig. 26)	**27**
25	Sensilla on maxillary palpus thick, foretarsal sensillum *a* half the length of *c* (see Szeptycki 2004: figs 48, 51)	**26**
–	Sensilla on maxillary palps slender, foretarsal sensilla *a* and *c* nearly equal in length (see Szeptycki 1985a: figs 66, 71)	***E. armatum***
26	Dorsal sensillum on maxillary palpus clearly longer than lateral sensillum (see Szeptycki 2004: fig. 48), length of foretarsus 95–100 μm	***E. noseki***
–	Sensilla on maxillary palpus nearly equal in length (see Szeptycki 2004: fig. 26), length of foretarsus 70–80 μm	***E. canarinum***
27	Foretarsal sensillum *c*’ in half distance between *α6* – *δ4*’, seta *P2a* on nota equal in length to *P3a* (see Szeptycki: figs 32, 37), length of foretarsus 65 μm	***E. umbrosum***
–	Foretarsal sensillum *c*’ closer to *δ4*’ than to *α6*, seta *P2a* shorter than *P3a* (see Rusek 1988: fig. 3B), length of foretarsus 75–85 μm	***E. stumppi***
28	Tergite VII with 10 *A*-setae (see Nosek 1973: fig. 31H)	***E. boedvarssoni***
–	Tergite VII with fewer *A*-setae	**29**
29	Tergite VII with 8 *A*-setae (*A3* absent) (see Aldaba 1986: fig. 17)	**30**
–	Tergite VII with 6 *A*-setae (*A1, A3* absent)	**31**
30	Sternites IX – X with 6 setae, female squama genitalis of “*wheeleri*” type (Nosek 1973: p. 95; Tuxen 1964: fig. 105)	***E. carolae***
–	Sternites IX – X with 4 setae, female squama genitalis of “*transitorium*” type (see Aldaba 1986: table 2, fig. 18)	***E. gamae***
31	Tergite VI with 10 *A*-setae	**32**
–	Tergite VI with 8 *A*-setae	**57**
32	Sternites IX – X with 4 setae	**33**
–	Sternites IX – X with 6 setae (sternite X with 4 setae in maturus junior)	**48**
33	Head with *aa* and *pa* setae (J. Rusek, pers. comm.; Arbea-Polite 1990: fig. 15a), seta *P1a* passing hind margin of tergite VII (see Nosek 1973: fig. 37H; Arbea-Polite 1990: fig. 7)	**34**
–	Head with *pa* setae or without additional setae	**35**
34	Foretarsal sensillum *t1* midway between *α3* and *α3*’, rostral seta evidently shorter than subrostral (see Nosek 1973: fig. 37B, C), length of foretarsus 86 μm	***E. gisini***
–	Foretarsal sensillum *t1* near to *α3*’, rostral and subrostral setae equal in length (see Arbea-Polite 1990: fig. 2a, 24), length of foretarsus 77–86 μm	***E. pinkyae***
35	Head without additional setae	**36**
–	Head with *pa* setae	**37**
36	Basal seta *D2* on hind leg about half the length of *D1* (see Szeptycki 1985b: fig. 85)	***E. rafalskii***
–	Basal seta *D2* on hind leg subequal with *D1* (see Szeptycki 1985b: figs 108, 109)	***E. silesiacum***
37	Basal seta *D2* on hind leg spine-like	**38**
–	Basal seta *D2* on hind legs setiform	**46**
38	Seta *P1a* not reaching hind margin of tergite VII	**39**
–	Seta *P1a* extending past hind margin of tergite VII	**42**
39	Sensilla on maxillary palps short and equal in length (see Szeptycki 1986: fig. 73, Szeptycki and Christian 2000: fig. 3)	**40**
–	Maxillary sensilla long, lateral sensillum longer than dorsal	**41**
40	Notal setae *P1* longer than *P1a*, foretarsal sensillum *f1* spatuliform (see Szeptycki 1986a: figs 74, 81)	***E. bryophilum***
–	Notal setae *P1* shorter than *P1a*, foretarsal sensillum *f1* filiform (see Szeptycki and Christian 2000: figs 4, 10)	***E. vindobonense***
41	Length ratio of notal setae *P1*:*P1a* ≥1.5 (see Szeptycki 1986a: fig. 31)	***E. enigmaticum***
–	Length ratio of notal setae *P1*:*P1a* ≤1.3 (see Szeptycki 1986a: fig. 46)	***E. gramineum***
42	Sensilla on maxillary palpus nearly equal in length (see Szeptycki 1986: fig. 158; Szeptycki and Christian 2000: fig. 24)	**43**
–	Lateral sensillum of maxillary palpus much longer than dorsal sensillum (see Szeptycki 1986a: figs 107, 123, 138)	**44**
43	Notal setae *P1a* and *P1* nearly equal in length (see Szeptycki 1986a: fig. 152), length of foretarsus 85–90 μm	***E. longisquamum***
–	Notal seta *P1a* shorter than *P1* (Szeptycki and Christian 2000: fig. 28), length of foretarsus 100–115 μm	***E. cetium***
44	Seta *P1a* on tergites I – VI longer than *P1*, foretarsal sensillum *t1* nearer to *α3* than to *α3*’, sensillum *t3* longer than *c*’, length of foretarsus less than 100 μm	**45**
–	Seta *P1a* on tergites I – VI equal in length or shorter than *P1*, foretarsal sensillum *t1* midway between *α3* and *α3*’ or slightly closer to *a3*’, sensillum *t3* short, equal in length to *c*’, length of foretarsus 100–110 μm (see Szeptycki 1986a: figs 115, 116)	***E. silvaticum***
45	Tracheal camerae long, slender; foretarsal sensillum *d* long, reaching base of *α6*, length of foretarsus 90–100 μm (see Szeptycki 1986a: figs 125, 126, 130, 131)	***E. semiarmatum***
–	Tracheal camerae short, stocky; foretarsal sensillum *d* short, not reaching base of *α5*, length of foretarsus 75–85 μm (see Szeptycki 1986a: figs 140, 145)	***E. parvum***
46	Seta *P1a* at level *P2* on tergite VII	**47**
–	Seta *P1a* slightly posterior to *P2* and extending past hind margin of tergite VII (see Szeptycki 1986a: fig. 63)	***E. posnaniense***
47	Rostral seta thinner than subrostral seta (see Szeptycki 1986a: fig. 6), lateral sensillum on maxillary palpus longer than dorsal sensillum (see Nosek 1973: fig. 28C’; Szeptycki 1986a: fig. 7), foretarsal sensillum *t1* nearer to *α3* than to *α3*’(see Nosek 1973: fig. 28A; Szeptycki 1986a: fig. 20) or midway between *α3* and *α3*’ (see Szeptycki 1986a: fig. 21)	***E. transitorium***
–	Rostral seta thicker than subrostral seta, sensilla on maxillary palpus nearly equal, foretarsal sensillum *t1* nearer *α3*’ than *α3* (see Szeptycki 1986a: figs 86, 100)	***E. mariae***
48	Seta *P1a* at level of *P2* on tergite VII	**49**
–	Seta *P1a* posterior to level of *P2* on tergite VII	**52**
49	Foretarsal sensillum *f1* thickened apically, body length more than 1600 μm (see Nosek 1973: fig. 32A; Rusek 1988: p. 229)	***E. mixtum***
–	Foretarsal sensillum *f1* not thickened apically, body length less than 1600 μm	**50**
50	Foretarsal sensillum *f1* thick, thicker than sensillum *a* (see Aldaba 1986: fig. 5), body length less than 850 μm	***E. lusitanicum***
–	Foretarsal sensillum *f1* thin (see Rusek 1988: fig. 8A), body length more than 1300 μm	**51**
51	Foretarsal sensillum *t1* midway between *α3* and *α3*’, sensillum *f2* sensilliform (see Rusek 1988: fig. 8A, B), body length about 1460 μm	***E. funkei***
–	Foretarsal sensillum *t1* nearer *α3* than *α3*’, sensillum *f2* spatuliform (see Nosek 1973: fig. 35A), body length about 1300 μm	***E. coiffaiti***
52	Head with both *aa* and *pa* setae	**53**
–	Head with *aa* or *pa* setae	**54**
53	Foretarsal sensillum *a* longer than half the length of *c*, sensillum *f1* spatulate (see Szeptycki 1984: fig. 38)	***E. mirabile***
–	Foretarsal sensillum *a* shorter than half the length of *c*, sensillum *f1* filiform (see Szeptycki 2001: figs 103, 105)	***E. weinerae***
54	Head with *aa* setae (see Stumpp and Szeptycki 1989: fig. 8)	***E. rusekianum***
–	Head with *pa* setae only	**55**
55	Lateral sensillum on maxillary palpus clearly longer than dorsal, spatulate dilation on foretarsal sensilla *e* and *g* short, about third of sensillum length, tracheal camerae long (see Szeptycki 1984: figs 7, 9, 11, 12)	***E. vulgare***
–	Sensilla on maxillary palps nearly equal in length, spatulate dilation on foretarsal sensilla *e* and *g* long, about half of sensillum length, tracheal camerae short and thickened (see Rusek 1988: fig. 6D; Szeptycki 1984: figs 20, 26, 27)	**56**
56	Foretarsal sensillum *a* about half of *c* length, lateral sensillum on maxillary palpus slightly shorter than dorsal (see Szeptycki 1986a: figs 20, 26)	***E. pinetorum***
–	Foretarsal sensillum *a* about equal in length to *c*, sensilla on maxillary palpus equal in length (see Rusek 1988: fig. 5A, 6D)	***E. fichteliense***
57	Sternites IX – X with 4 setae	**58**
–	Sternites IX – X with 6 setae, head with *pa* setae (see Nosek 1973: fig. 32F’; Szeptycki 1984: p. 200)	***E. germanicum***
58	Head without additional setae (Szeptycki 1985b: p. 532), length of foretarsus 100–110 μm	***E. sudeticum***
–	Head with *pa* setae, length of foretarsus ≤85µm	**59**
59	Head with both *aa* and *pa* setae (see Szeptycki 1999: fig. 1), length of foretarsus 80–85 μm	***E. ulinense***
–	Head with only *pa* setae (see Szeptycki 1993: fig. 43), length of foretarsus 70–80 μm	***E. solarzi***
